# Size compensation in *Drosophila* after generalised cell death

**DOI:** 10.3389/fcell.2023.1301913

**Published:** 2023-11-21

**Authors:** Noelia Pinal, Mireya Ruiz-Losada, Natalia Azpiazu, Ginés Morata

**Affiliations:** ^1^ Departamento de Odontología Pre-clínica, Universidad Europea de Madrid, Villaviciosa de Odon, Spain; ^2^ Centro de Biología Molecular, CSIC-UAM, Madrid, Spain

**Keywords:** cell death, irradiation, size compensation, JNK, WG, JAK/STAT

## Abstract

Regeneration is a response mechanism aimed to restore tissues that have been damaged. We are studying in the wing disc of *Drosophila* the regenerative response to a dose of Ionizing Radiation that kills over 35% of the cells distributed all over the disc. After such treatment the discs are able to restore normal size, indicating there is a mechanism that repairs generalised damage. We have tested the role of the JNK, JAK/STAT and Wg pathways, known to be required for regeneration after localised damage in the disc. We find that after irradiation there is size compensation in the absence of function of these pathways, indicating that they are not necessary for the compensation. Furthermore, we also find that generalised damage does not cause an increase in the proliferation rate of surviving cells. We propose that irradiated discs suffer a developmental delay and resume growth at normal rate until they reach the final stereotyped size. The delay appears to be associated with a developmental reversion, because discs undergo rejuvenation towards an earlier developmental stage. We argue that the response to generalized damage is fundamentally different from that to localized damage, which requires activity of JNK and Wg.

## Introduction

Animal tissues react to developmental insults (amputation, injury, irradiation) regenerating the damaged or missing parts through a process that includes compensatory proliferation and reprogramming of the resident cells that survive the damage (Alvarado, 2000; Worley et al., 2012).

We are studying the mechanisms of regeneration using the *Drosophila* wing imaginal disc as a model system. This disc is a classical object of research in developmental biology; its growth parameters are well known as well as the different genetic factors and signalling molecules involved in its development (see review in (Tripathi and Irvine, 2022)). It begins growth at the interphase between first and second larval instar with about 50 cells to reach a total of 31,000 cells at the end of the larval period (Martín et al., 2009a). On average the progeny of each initial cell performs 9–10 divisions. It is of interest that the cell division rate is not uniform during the proliferation period; cells proliferate rapidly during the early stages, about 5–6 h per cycle, but the rate decreases later in development to about 30 h per cycle at late third instar (Martín et al., 2009b). The disc stops growth at the end of the larval period, at the time pupation starts.

The wing disc has strong regenerative potential; disc fragments have been shown to regenerate an entire disc after transplantation into adult hosts (Bryant, 1971; Schubiger, 1971). More recent experiments (Smith-Bolton et al., 2009; Bergantiños et al., 2010; Herrera et al., 2013; Martín et al., 2017) also demonstrate strong *in vivo* regeneration. The common approach is to make use of the Gal4/UAS method to target a particular region of the disc for ablation and then to study how the reminder of the disc reacts and reconstitutes the damaged part. This method allows a precise definition of the target region to ablate. Those experiments have identified several major factors, the Jun N-Terminal Kinase (JNK), Wingless (Wg) and Myc pathways, which are implicated in the process.

The activity of JNK appears to be central to the process: 1) it becomes activated in cells suffering damage, where it triggers apoptosis and subsequent cell death (McEwen and Peifer, 2005), and 2) JNK-expressing cells secrete mitogenic signals that stimulate the proliferation of neighbour cells—a paracrine function (Pinal et al., 2019). We have shown that these two functions operate in regenerating wing discs: in an experiment in which wing cells are killed, the moribund JNK-expressing cells release signals that induce additional proliferation of neighbour notum cells, which generate a notum duplicate. If JNK signalling is prevented the duplicated notum does not appear (Martín et al., 2017).

Unlike the experiments above in which the damage is restricted to specific disc regions, treatments like ionising irradiation (IR) cause pepper-salt generalised damage, killing a fraction of cells thorough the entire territory of the disc. Published reports indicate that for a dose of 4000R the wing disc can compensate for the loss of 50% or more of the cell population and subsequently attains normal final size (Jaklevic and Su, 2004).

The mechanism(s) by which the size of the cell population is reconstituted after generalised cell death are unclear. The mode of function of JNK provides an attractive model: as described above, it is activated after IR in cells fated to die but also elicits the secretion of mitogenic signals, which are received by healthy neighbours. We have shown that this mechanism plays a critical role in the regeneration of wing disc fragments (Martín et al., 2017).

The same mechanism could operate in the case of generalised cell death; JNK activation in the dying cells would provide proliferative signals to healthy cells in the proximity, which would perform additional divisions to compensate for the cell loss. Some years ago, we showed that two downstream JNK signals, Dpp and Wg, are not involved (Pérez-Garijo et al., 2009) in size proliferation, but there is evidence that JNK can activate the Janus Kinase and signal transducer and activator of transcription proteins (JAK/STAT) pathway (Wu et al., 2010; Pinal et al., 2018), known to induce cell proliferation in imaginal discs (La Fortezza et al., 2016) and there is also the possibility of other downstream signals.

Moreover, it has been proposed (Verghese and Su, 2016) that in the wing disc much of the size compensation after IR derives from cells of the proximal region of the disc (the hinge), which are refractory to radiation-induced apoptosis: cells from the hinge can migrate to the central region to replace the lost ones. The insensitivity of those cells to apoptosis requires activity of the JAK/STAT and Wg pathways, which implies that both pathways are necessary for the compensation. This hypothesis is supported by lineage data showing that clones from the hinge can extend to the pouch (Verghese and Su, 2016), although it does not explain size compensation in other parts of the disc like the notum.

Thus, there are three pathways that could be involved in the process of size compensation by inducing additional proliferation of cells surviving irradiation: JNK, JAK/STAT and Wg. Here we analyse the role of these pathways in irradiated wing discs that lose at least 35% of their cells. We describe their response to IR and also test whether their activity is necessary for compensating for the cell loss. Our principal finding is that irradiated wing discs achieve normal size in the absence of function of JNK, JAK/STAT or Wg. We propose that size compensation after generalised cell death does not require a specific upsurge of compensatory proliferation but that the disc responds to the loss of cells by rejuvenating, thus prolonging the proliferative period to allow for additional cell divisions until it reaches the final stereotyped size. These results indicate that the response of the wing disc after generalised damage is fundamentally different from that after localised damage.

## Results

### Amount of apoptosis and recovery of the wing disc after irradiation

**FIGURE 1:**
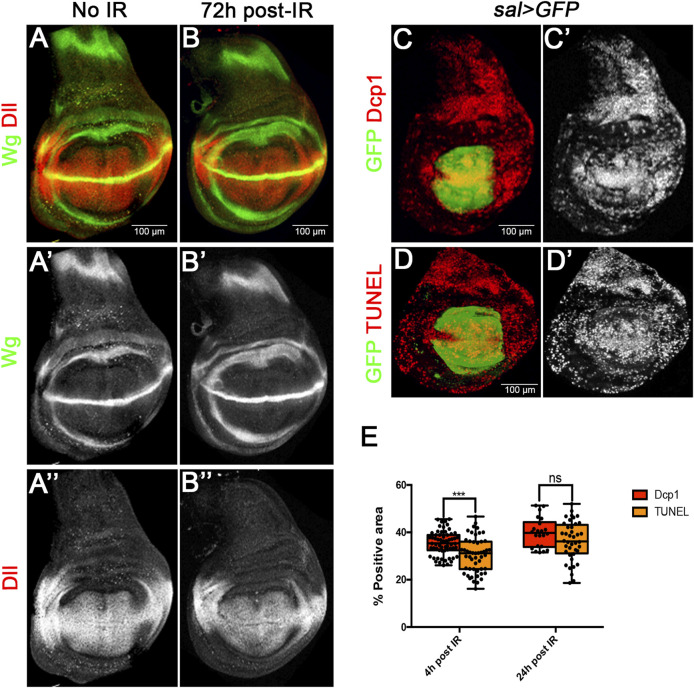
Size restoration and apoptosis after Ionizing irradiation (IR). **(A–B)** Confocal images of wild type wing imaginal discs of L3 wandering larvae showing Wg (green) and Dll (red) patterns in non-irradiated larvae as control **(A–A”)**, and in larvae 72 h after irradiation with 3000R **(B–B”)**. The final size and Wg and Dll expression are fully restored in irradiated discs. **(C–E)** Quantification of apoptosis in *sal > GFP* wing discs after irradiation. **(C,D)** Examples of confocal images of wing discs 24 h after irradiation used for the quantification of apoptosis. GFP (green) labels all the cells of the Sal domain, whereas Dcp1 (C-C′) or TUNEL (D-D′) are labelled in red/white. **(E)** Graph showing the percentage of Dcp1 or TUNEL staining within the GFP area 4 h or 24 h after irradiation. This value reflects the percentage of cells that are killed by the treatment.

The ability of imaginal discs to recover after IR has been known for a long time. It is illustrated in Figures 1A, B: larvae administered a heavy dose (3000R) of X-rays at second or early third larval period are able to generate mature (prepupal) wing discs indistinguishable from those of non-irradiated larvae. This indicates that the size compensation has occurred during the larval period. Mature irradiated discs also show normal expression of the developmental gene *wingless* and its target *Distalless* (Dll) Figures 1A, B).

To measure the cell lethality caused by IR in our experiments, we irradiated larvae of *sal > GFP* genotype and fixed wing discs 4 and 24 h after the treatment. All the cells of the Sal domain (which occupies the central region of the disc, Figures 1C, D) are labelled with GFP. We then estimated the percentage of cells of the domain which contain Caspase activity or TUNEL staining with respect to the total of GFP labelled cells. The percentage provides a measurement of the amount of the cell death caused by the irradiation.

The results are shown in Figure 1E. 4 h after the irradiation 35% of the cells show Dcp1 and 30% TUNEL staining. This difference is small but significant and suggests that some of the cells that gain Dcp1 activity do not complete the apoptotic process. 24 h after IR the proportion of Dcp1 and TUNEL cells has raised to 39% and 36% respectively. At this time the difference between Dcp1 and TUNEL is not significant, indicating that all Dcp1 labelled cells die. We estimate that an IR dose of 3000R causes over 35% of cell death, although this is very likely an underestimate because there is still cell death even 48 h after the irradiation (Pérez-Garijo et al., 2004).

### The JNK, JAK/STAT and Wg pathways after irradiation

**FIGURE 2:**
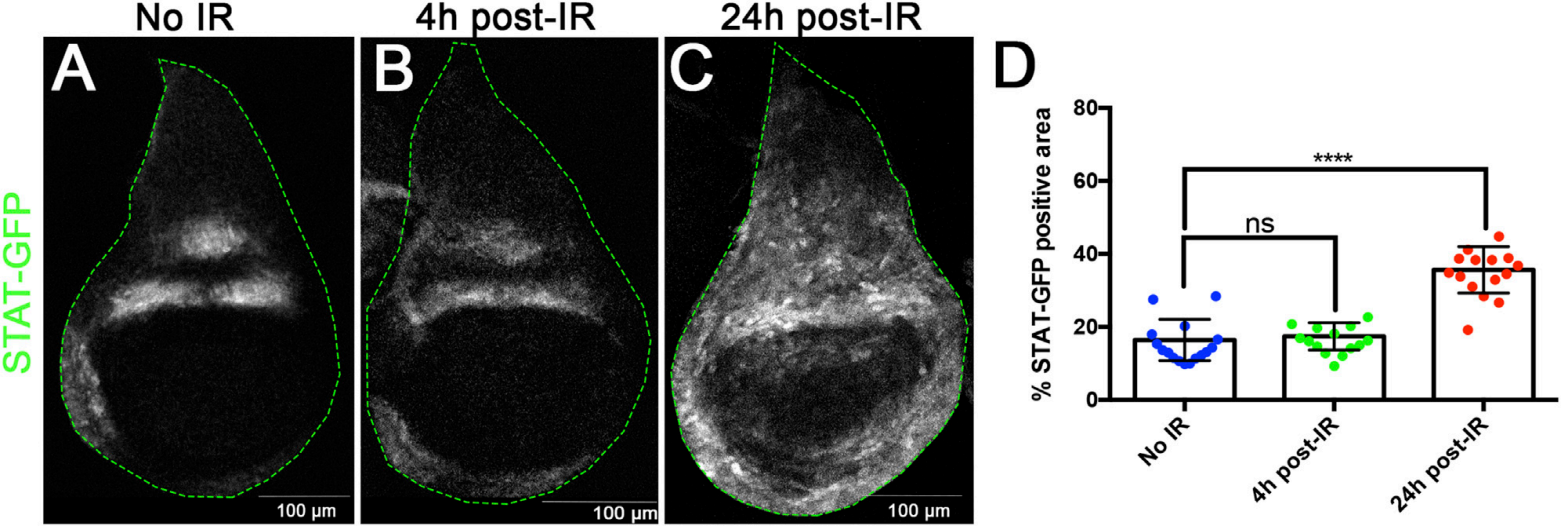
X-radiation induces ectopic JAK/STAT activity. **(**A–C) Confocal images of wildtype wing discs showing expression of the *STAT-10xGFP* reporter as indicator of JAK/STAT activity. **(A)** non-irradiated, **(B)** 4 h after irradiation, **(C)** 24 h after irradiation. **(D)** Graph showing the quantification of STATGFP expression in the pouch region in the control and 4h and 24 h after IR. There is upregulation of JAK/STAT activity in the wing pouch after IR, which is clearly significant after 24 h.

The idea that JNK, JAK/STAT and Wg could be implicated in size compensation after generalised cell death is supported by their response to IR. It is known that JNK and Wg are triggered upon irradiation (Pérez-Garijo et al., 2009; Wu et al., 2010; Pinal et al., 2018): both pathways become de-repressed in regions where they are not normally active. In addition, we have examined the expression of JAK/STAT after IR and find it is also upregulated; it is normally restricted to the hinge region of the disc (Ayala-Camargo et al., 2013), but after IR there is gain of expression in the wing pouch area, clearly detectable after 24 h (Figure 2). This upregulation of JAK/STAT is not surprising since we have previously shown that after irradiation it functions downstream of JNK (Pinal et al., 2018). However, it has been reported (Beira et al., 2018) that it can function independently of JNK.

#### Suppression of JNK, JAK/STAT and Wg in irradiated P compartments

The upregulation of JNK, Wg and JAK/STAT upon IR of the wing disc ((McEwen and Peifer, 2005; Pérez-Garijo et al., 2009; Pinal et al., 2018) and Figure 2) suggested that these pathways would be involved in the regenerative response; their function would stimulate cell proliferation of surviving cells as to compensate for the cell loss. In absence of their function the process of size compensation would be compromised.

**FIGURE 3:**
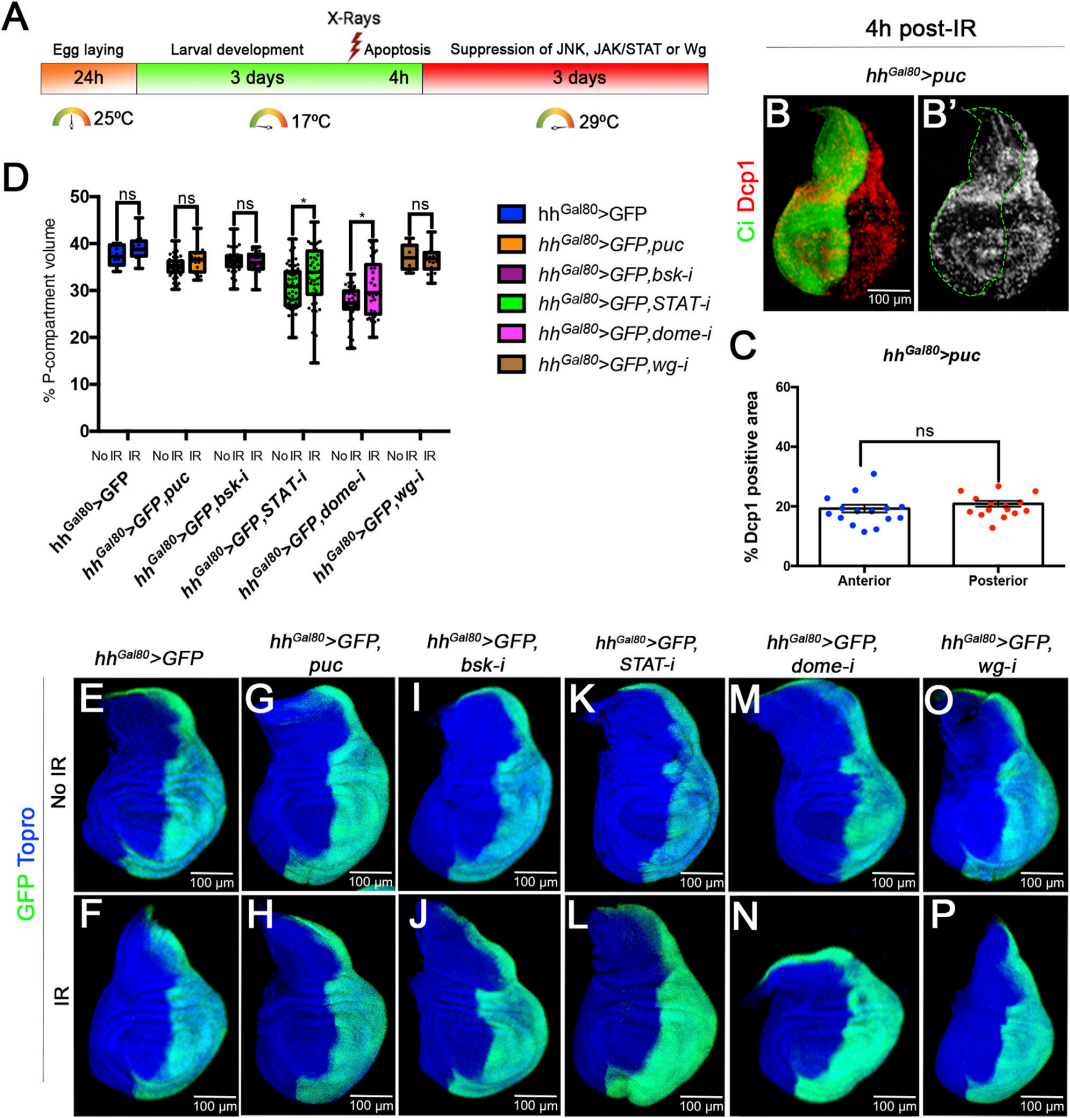
Size compensation in the absence of Wg, JAK/STAT or JNK activity. **(A)** Schematic representation of the experiment used to assay the role of Wg, JAK/STAT and JNK pathways in size compensation. Larvae of the genotype indicated in the figure were kept at 17°C for 3 days after egg laying before IR. After irradiation they were kept for 4 h at 17°C to allow apoptosis and then switched to 29°C to induce the expression of the UAS transgenes that suppress the function of JNK, JAK/STAT or Wg (see Material and Methods for details). The larvae were maintained at 29°C for the rest of development until the wandering stage and then dissected and wing discs fixed. **(B,B’)** Confocal image of a third instar disc of the genotype indicated stained for apoptosis (Dcp1, red/white) 4 h after IR. The anterior compartment is labelled green with the Ci antibody. Note that the amount of apoptosis, monitored by Dcp1 (red/white), is the same in the anterior and posterior compartment. **(C)** Quantification of the levels of apoptosis 4 h after irradiation in of disc with genotype describe in **(B)**. The percentage of Dcp1 stain is similar in A and P compartments. **(D–P)** Examples of confocal images of wing discs used for the quantification, GFP (green) labels the posterior compartment, and TOPRO-3 (blue) the entire disc. **(D)** Graph showing the volume of the posterior compartment with respect to the total volume of the disc in the different genotypes studied. There are not significant differences in posterior compartment volume between irradiated and no-irradiated larvae for all the cases. The discs in which the JAK/STAT pathway is suppressed the posterior compartments are smaller, but in both irradiated and no-irradiated discs. **(E–P)** Confocal images of example imaginal discs used for the quantification. Non-irradiated discs upper images, irradiated ones lower images. **(E,F)** control discs. **(G–J)**; discs in which JNK pathway is supressed in the posterior compartment by the expression of **(G,H)** *puc* or **(I,J)** *UAS-bskRNAi*; **(K–N)** discs in which JAK/STAT pathway is supressed in the posterior compartment by **(K,L)** *UAS-STATRNAi* and **(M,N)** *UAS-DomeRNAi*; and **(O,P)** discs in which Wg pathway is supressed in the posterior compartment by *UAS-wgRNAi*,.

To assay their role in size compensation after IR we have made use of the Gal4/UAS Gal80^TS^ system (Figure 3A) to suppress the activity of each of these pathways in the posterior (P) compartment by using effective UAS-RNAi lines that inhibit their function. If size compensation after IR in the posterior compartment is abolished the percentage of P tissue in the disc should be significantly decreased.

Suppression of JNK activity can be achieved by RNA interference of the Basket kinase (Wang et al., 2015) or overexpressing *puckered*, which functions as a negative regulator of JNK (Martín-Blanco et al., 1998; McEwen and Peifer, 2005). The JAK/STAT pathway can be inactivated by suppressing the function of the transcription factor STAT (Verghese and Su, 2016) or the receptor Domeless (Brown et al., 2001), and the Wg pathway by interfering with the ligand itself (see *Material and Methods*). Those UAS transgenes have been shown to be effective suppressors of the corresponding pathways (Verghese and Su, 2016; Martín et al., 2017).

**FIGURE 4:**
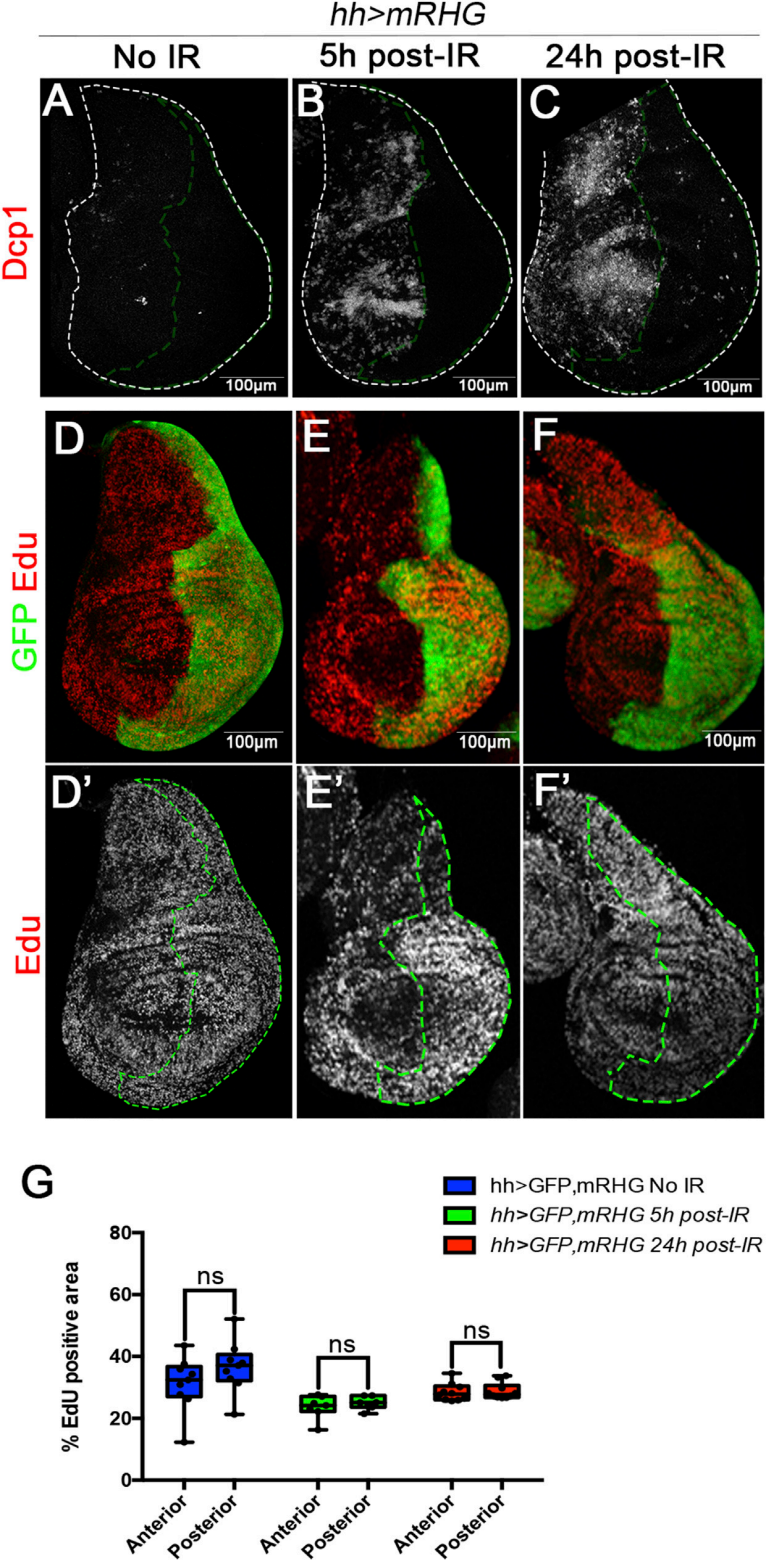
Massive cell loss by IR does not trigger immediate upsurge of cell division. **(A–C)** Confocal images of wing discs of genotype *hh > GFP RHGmiRNAi*, in which the apoptosis is suppressed in the posterior compartment by the activity of the *UAS-RHGmiRNAi* construct that abolishes the function of the *reaper*, *hid* and *grim* pro-apoptotic genes. **(A)** Control no-irradiated disc stained with Dcp1 (red); **(B,C)** discs 5h and 24 h after IR, there are high levels of apoptosis (Dcp1, red) in the anterior compartment but virtually none in the posterior compartment, indicating that there is no cell loss. **(D,D’)** non-irradiated disc showing similar levels of EdU incorporation (red/white) in the anterior and posterior (labelled with GFP) compartments. **(E–F’)** EdU incorporation in the anterior and posterior compartment 5h and 24 h after irradiation. **(G)** Graph showing quantification of EdU incorporation in the anterior or posterior compartments 5 and 24 h after irradiation in discs of the genotype above. It indicates that the loss of cells in the anterior compartment does not induce an upsurge of cell proliferation shortly after irradiation.

Since normal function of JNK is necessary for induction of apoptosis (McEwen and Peifer, 2005), the analysis of its possible contribution to size compensation upon IR requires that JNK be suppressed only *after* cell death has occurred. To address this issue, we have used the thermosensitive dominant Gal4 suppressor Gal80^TS^ to manipulate the time of activity of JNK. Larvae of genotype *UAS-GFP; TubGal80* ^*TS*^ *hhGal4; UAS-puc* (or *UAS-bskRNAi*) were irradiated at the second/early third period (3 days at 17°C after egg laying) and shifted to 29°C 4 h after IR, thus providing normal JNK function for those 4 h plus the time needed to inactivate the Gal80 suppressor, estimated to be about 6 h^26^. We certified that there is apoptosis in those experimental conditions by measuring the levels of Dcp1 4 h after IR of third instar discs of genotype *hh > GFP UAS-puc TubGal80* ^*TS*^ grown at 17°C and before the shift to 29°C. As illustrated in Figures 3B, C, 4 h after IR at 17°C we find similar levels of apoptosis in the A and P compartments.

To assay the requirements for JAK/STAT or Wg we used a similar protocol (see Material and Methods). The results concerning the requirements of the three pathways for size compensation are shown in Figures 3D–P. In comparison with controls (Figures 3E, F) the size of the P compartment is not reduced after compromising the JNK (Figures 3G–J), JAK/STAT (Figures 3K–N) or Wg (Figures 3O, P) pathways. The quantified results shown in Figure 3D indicate that the percentage of P compartment with respect to the total volume of the disc is in all cases like in the control non-irradiated discs. In the case of the suppression of JAK/STAT we find that the non-irradiated STAT RNAi or Dome RNAi P compartments are smaller than the normal ones (Figure 3D), suggesting it is required for normal growth of the compartment. Intriguingly, in irradiated discs in which JAK/STAT is suppressed the posterior compartment grows slightly more than in non-irradiated ones.

The conclusion from the preceding experiments is that in the absence of JNK, JAK/STAT or Wg, irradiated discs compensate for the loss of 35% of their cells and attain normal size and shape. These results strongly suggest that these pathways do not play a significant role in size compensation after generalised cell death.

A caveat of the previous interpretation is the possibility of an immediate and strong proliferative response, dependent on JNK or on the two other pathways, within the few hours between the time of irradiation and the suppression of the pathway by Gal80. If that were the case, we would expect a significant augment of the expression of cell division markers like EdU or PH3 in the affected region just after IR.

We have tested this possibility by measuring EdU incorporation 5 h after IR of discs of genotype *hh > GFP UAS-RHGmiRNA.* In this experiment the *UAS-RHGmiRNA* construct suppresses the function of *reaper*, *hid* and *grim*, the three principal pro-apoptotic genes and is a very effective suppressor of apoptosis (Siegrist et al., 2010). As shown in Figures 4A–C, IR does not trigger cell death in the P compartment and hence there would be no need for compensatory proliferation, which would be needed in the A compartment. An upsurge of cell proliferation in the A compartment after IR should be reflected in higher incorporation levels of cell division markers like EdU or PH3 in comparison with the P compartment. As illustrated in Figures 4D–F’, EdU levels 5 and 24 h after IR are the same in the A and the P compartment. These results are quantified in Figure 4G. These results rule out the possibility of an immediate proliferation response to IR.

### Developmental response after IR

Our results indicate that the activation JNK, JAK/STAT or Wg pathways after IR does not prompt a specific increase of growth rate to compensate for generalised cell loss. It is intriguing because those pathways are major factors involved in growth, regeneration and patterning of the wing disc (reviewed in (Tripathi and Irvine, 2022)). They are also required for the regenerative response to damage to localised damage to the disc (Smith-Bolton et al., 2009; Bergantiños et al., 2010; Santabárbara-Ruiz et al., 2015).

An alternative is that generalised cell death induced by IR, unlike localised damage, does not trigger an upsurge proliferative response. The process would be as follows: 1) IR kills a large fraction of the cell population, 2) the affected disc becomes smaller than non-irradiated controls and may revert to an earlier developmental stage, 3) the smaller disc resumes growth at the normal rate until it reaches the final stereotype size. This process prolongs the proliferation period of the disc. This model proposes that irradiated discs would revert to an earlier developmental stage and would grow at normal rate, but for a longer time; we refer to it as developmental compensation.

There are two aspects of this model that we have tested: 1) the proliferation rate is not affected by the loss of cells caused by the irradiation, and 2) the irradiated discs revert to an earlier developmental stage.

#### Cell proliferation after IR

**FIGURE 5:**
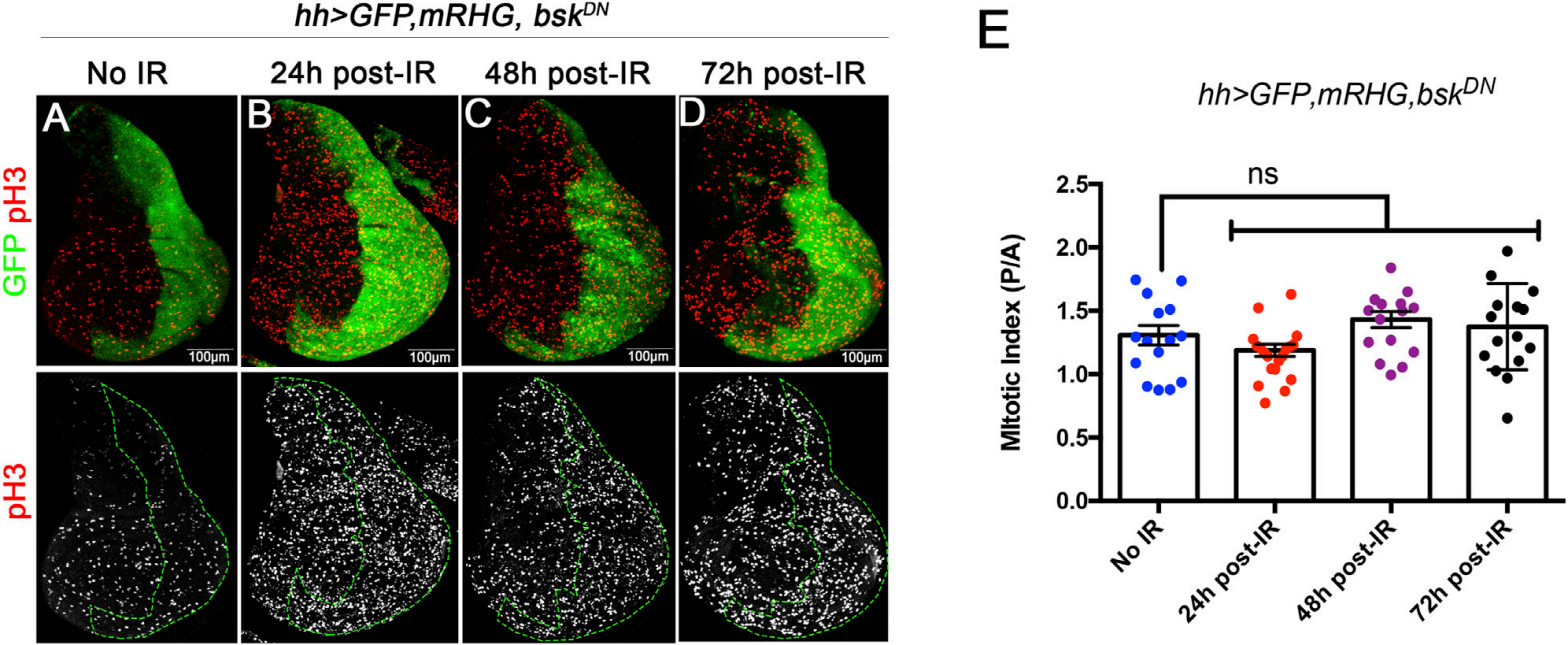
Mitotic index measured by the number of cells stained with PH3 by surface area, at 24, 48 and 72 h after IR in discs of *hh > UAS-GFP UAS-miRHG UAS-bsk* ^*DN*^ genotype. **(A–D)** examples of discs illustrating the PH3 (red) staining levels at different time points are the same in the anterior and posterior (labelled green) compartment. **(E)** Graph showing quantification of the MI ratio between posterior (P) and anterior (A) compartments in control no-irradiated discs and discs 24, 48 and 72 h after irradiation.

We have carefully measured the cell proliferation rates during development of anterior and posterior compartments after irradiation of discs of genotype *hh > miRHG bsk* ^*DN*^. In these discs the cells in the posterior compartment cannot enter apoptosis as they are deficient in the activity of the pro-apoptotic genes. They are also deficient in JNK activity, what prevents the overgrowths caused by the ectopic activation of JNK in apoptosis-deficient cells (Pinal et al., 2018). Under these circumstances IR will kill at least 35% of the cells of the A compartment, whereas there will be no cell death in the P compartment. Using PH3 as marker of cells in mitosis we have measured the mitotic index (MI, number of PH3 dots per surface units in µm^2^) of the Anterior and Posterior compartments of discs 24, 48, 72 h after IR. The results are shown in Figures 5A–E: the ratio of MI in the A and P compartment is the same irrespective of the time after IR. They strongly suggest that the growth rate is not altered by the massive cell death caused by IR.

#### Irradiated discs revert to an earlier developmental stage

The possibility that irradiated discs revert to an earlier stage can be tested by examining the expression pattern of the genes *wingless (wg) and Distalless (Dll)*, known to evolve in the wing disc during larval development (Martín and Morata, 2006).

To ensure little dispersion in the developmental stage of the larvae we performed the following procedure (see further details in the Material and Methods section): 96 h after a short egg-laying period of 2–3 h, some larvae were irradiated and their non-irradiated sibs dissected and the wing discs fixed for staining with anti-Wg and anti-Dll antibodies. The irradiated larvae were allowed to develop for 8 or 24 h after IR and their discs fixed and stained with the same antibodies.

**FIGURE 6:**
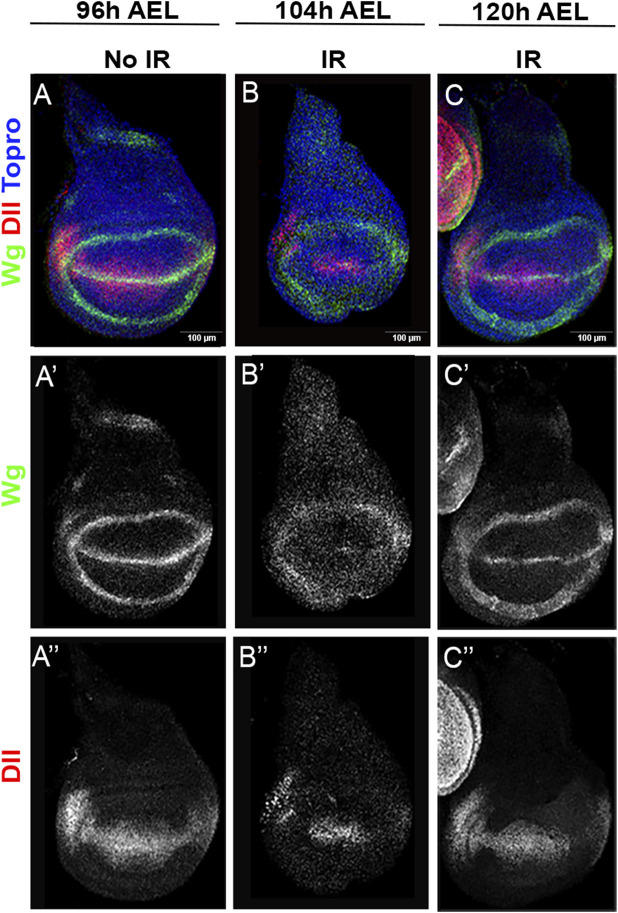
*wg* and *Dll* expression after IR. **(A–A”)** wildtype wing disc fixed 96 h after egg laying (AEL), which corresponds to middle third instar. It has been stained for *wg* [green or white **(A’)**] and *Dll* [red or white **(A”)**] expression with specific antibodies. **(B–B”)** Disc of the same genotype irradiated at 96 h (AEL) showing *wg* **(B’)** and *Dll* expression **(B”)** 8 h after IR. **(C–C”)** Disc of the same genotype irradiated at 96 h (AEL) showing *wg* and *Dll* expression 24 h after IR.

Figure 6 illustrates the differences in *wg* and *Dll* expression in irradiated and non-irradiated discs. While at 96 h in non-irradiated controls the expression patterns of *wg* and of *Dll* are well defined (Figures 6A–A”), the experimental discs show 8 h after irradiation (104 h of development) that these patterns are less defined (Figures 6B–B”), suggesting that they have reversed to a developmental stage earlier than that corresponding to 96 h of development (Martín and Morata, 2006). 24 h after IR the experimental discs exhibit more mature *wg* and *Dll* expressions (Figures 6C–C”), very similar to those of control 96 h discs.

We have also examined the expression pattern after IR of genes that become active during late developmental stages. We have chosen the genes *cut*, *senseless* (*sens*), and *scute* (*sc*), which are expressed and required in the sensory organ precursors (Cubas et al., 1991; Blochlinger et al., 1993; Nolo et al., 2000; Martín and Morata, 2006). The activities of these genes can be considered as biomarkers of sensory organs.

**FIGURE 7:**
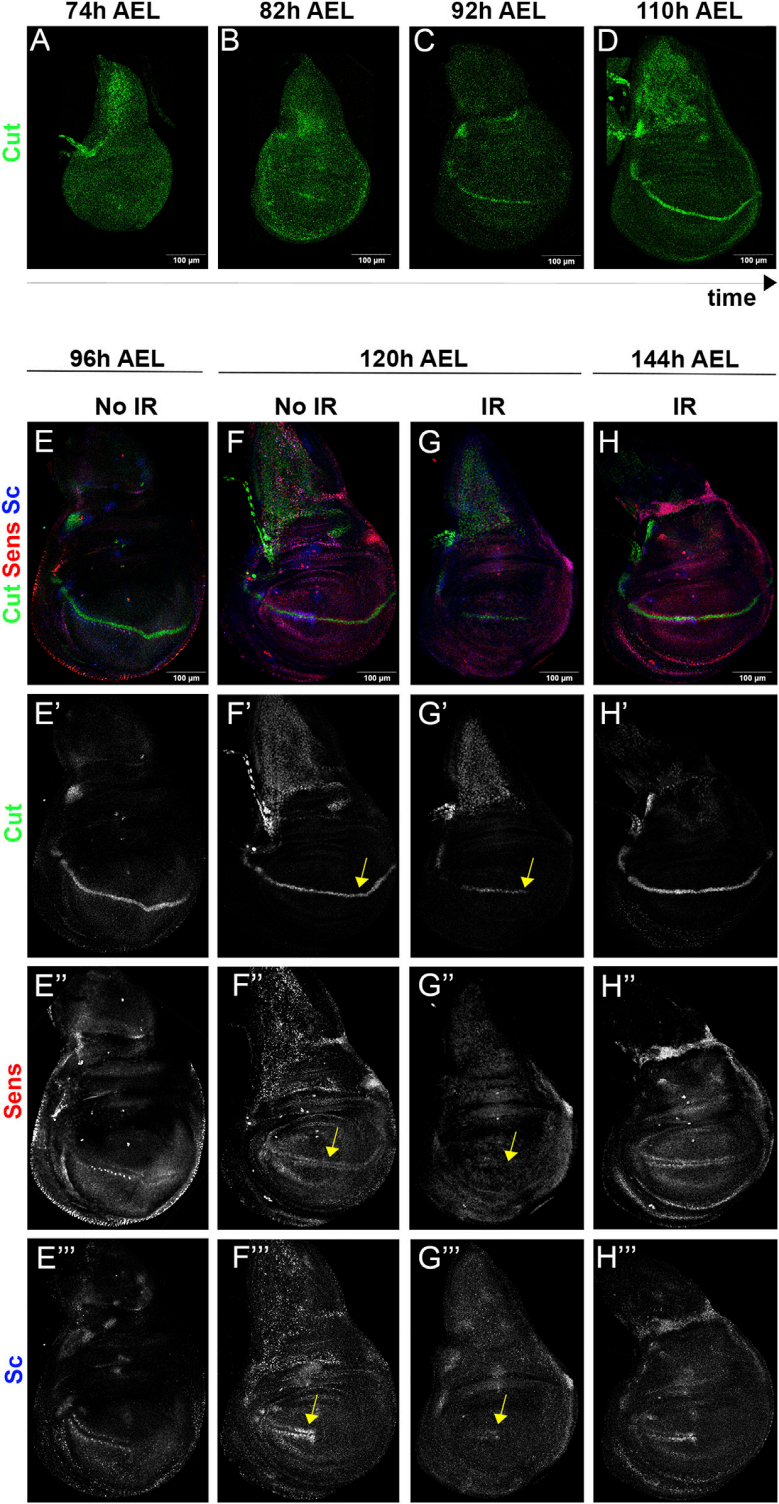
The wing disc becomes rejuvenated after IR. **(A–D)** Expression of *cut* in the wing disc at different moments during third instar. At 74 h after egg laying (AEL) the expression is diffuse, but at later times becomes gradually focalised in the dorso/ventral compartment boundary. **(E–H”’)** triple staining of *cut* (green), *sens* (red) and *sc* (blue) in irradiated and non-irradiated discs. The expression of the genes in 96 h AEL discs is shown in the left column **(E–E”’)**. The two middle columns show their expression at 120 h AEL in non-irradiated **(F–F”’)** and irradiated **(G–G”’)** discs. Note that the pattern of expression of *cut* **(G’)** *sens* **(G”)** and *sc* **(G”’)** in irradiated discs appears to correspond to a developmental stage earlier than 96 h (compare with the corresponding images in the **(E,F)** columns, indicated by yellow arrows). In irradiated 144 h AEL discs [**(H)** column] the expression of the three genes corresponds to a mature stage.

The results are shown in Figure 7. Because the evolution of the expression of *cut* during the last stages of the wing disc development had not been reported we describe it in Figures 7A–D. The expression of *cut*, *sens* and *scute* was examined by triple staining (Figure 7E–H”’) using specific antibodies.

In mature discs *cut* is strongly expressed along the dorso/ventral boundary (Blochlinger et al., 1993) (see also our Figure 7D), but the expression is weaker in younger discs of 92 h and rudimentary in 82 h AEL discs (Figures 7B, C). Earlier stages do not show *cut* expression (Figure 7A). We have examined the effect of IR on *cut* expression, illustrated in Figure 7E’–H’. Non-irradiated 96 h AEL discs already show a clear line of expression along the D/V border (Figures 7E’–F), but the expression of *cut* in the irradiated siblings 24 h later (120 h AEL) exhibit less advanced expression (Figure 7G’, arrow), corresponding to a developmental stage earlier than 96 h.

Nonetheless, 48 h after IR (144 h AEL) the irradiated discs attain the expression of *cut* corresponding to a mature disc (Figure 7H’).

We find similar results with *sens* and *sc*. In non-irradiated control discs at 96 and 120 h AEL *sens* is expressed in the sensory organ precursors along the entire wing margin (Nolo et al., 2000) (Figures 7E”, F”), and *sc* is expressed in the precursors of the anterior wing margin (Cubas et al., 1991) (Figures 7E”’, F”’). These observations indicate that by 96 AEL of normal development the prospective pattern of the of the triple row bristles of the wing margins is already established. We find that in 120 h irradiated discs the expression of *sens* and *sc* in the precursors of the tripe row is lost (Figures 7G”, G”’, arrows), indicating a regression to an earlier developmental stage, in which the triple row pattern is not yet established. By 144 h AEL the irradiated discs show the expression of both genes in the precursor of the triple row, indicating that the patterning process has been reiterated.

All together the results about the alterations after IR in the expression of *wg*, *Dll* and the sensory organs genes strongly suggest that irradiated discs revert to an earlier developmental stage. The fact that by 48 h (144 h of development) after irradiation all the sensory organs biomarkers have reappeared indicates a final restoration of the pattern after the delay caused by the rejuvenation of the disc.

## Discussion

When analysing regeneration process, we can distinguish between the response to localised damage, e.g., amputation or local injury, with that observed after generalized damage caused by irradiation. The response to localised damage has been analysed in a number of publications (Smith-Bolton et al., 2009; Bergantiños et al., 2010; Herrera et al., 2013). Key elements in the response are the JNK and Wg pathways, necessary for the additional proliferation required and possibly for the change of identity of the cells involved in the regeneration. It has been shown that during the regeneration process proliferative signalling emanating from dying JNK-expressing cells stimulates the proliferation of neighbour cells (Martín et al., 2017).

In the experiments reported here we have analysed the response of the wing disc to IR-induced generalised cell death, which eliminates a minimum of 35% of the population. Survivor cells compensate for the lost ones and eventually develop a disc of normal size and shape. Because of its mode of function, the JNK pathway would be expected to be involved in the compensation process; its activation by IR causes cell death by apoptosis, but dying JNK-expressing cells secrete signals that induce proliferation of neighbour cells (Martín et al., 2017) and reviewed in (Pinal et al., 2019). This paracrine activity would be responsible for the compensation. Previous work from our laboratory (Pérez-Garijo et al., 2009) suggested that there is size compensation in the absence of the JNK downstream signals Wg and Dpp, but there are other growth factors, directly or indirectly dependant on JNK, that could be responsible for the compensation. The experiments reported here rule out a role of JNK in the process as there is size compensation in the absence of JNK function (Figure 3).

Nonetheless, there might be other factors involved in the process. The JAK/STAT pathway is involved in the regeneration response to injury (La Fortezza et al., 2016). Moreover, in experiments reporting the response of the wing disc to irradiation Verghese and Su (Verghese and Su, 2016) described a proximal region of the disc (that they refer to as the “frown”), which contains high expression of *wg* and JAK/STAT and that is refractory to IR-induced apoptosis. The insensibility of the frown cells to apoptosis requires the function of the JAK/STAT and Wg pathways, for in their absence the amount of apoptosis after IR is like in the rest of the disc. Based on these observations, Verghese and Su (Verghese and Su, 2016) proposed that the cells from the frown are responsible for repopulating the disc after IR, and also provided some supporting evidence based in cell lineage, as clones of cells that originate in the frown region can extend to the wing pouch. However, the clones originating in the frown do not appear to extend to the notum, thus their model could only apply to the wing pouch.

An implication of the model by Verghese and Su (Verghese and Su, 2016) is that the ability of the frown cells to repopulate the rest of the disc after IR is dependent on JAK/STAT and Wg. However, our experiments demonstrate that there is size compensation in the absence of either Wg or JAK/STAT activity, arguing against the involvement of the cells of the frown.

The *p53* gene has been postulated to play a role in size compensation after IR (Wells and Johnston, 2012). IR-treated larvae suffer a slow-down in development, which requires *p53* function, and it has been suggested (Wells and Johnston, 2012) that it is necessary for the recovery of IR-treated larvae, because in *p53* mutant larvae the delay does not occur and the adults that emerge have small wings and multiple morphological abnormalities. However, irradiated *p53* mutant discs attain at the end of larval development the same size as normal discs (Wells and Johnston, 2012) indicating that *p53* is not required for size compensation. In *p53* mutants the temporal suppression of apoptosis may allow aneuploidy and other damaged cells to survive, which may be responsible for the abnormalities observed in IR-treated *p53* mutant flies.

The general conclusion from all the above is that none of the pathways examined, JNK, JAK/STAT, Wg or p53, is responsible for the size compensation after IR. This is in striking contrast with the response observed after localised damage in which JNK and Wg pathways are essential (Smith-Bolton et al., 2009; Bergantiños et al., 2010; Martín et al., 2017; Pinal et al., 2019) and indicates that the regenerative response mechanisms triggered by localised or by generalised cell death are fundamentally different.

### Proliferation after generalised damage

Our results indicate that in the wing disc, and by extension in the other discs, the mechanism to restore size and shape after generalised damage is very different from that operating after localised damage or amputation.

A localized damage like the ablation of the wing pouch or of other regions of the disc causes upregulation of the JNK pathway (Bosch et al., 2005; Smith-Bolton et al., 2009; Bergantiños et al., 2010; Herrera et al., 2013), which is a major factor in the response: it causes death of the damaged cells by activating the pro-apoptotic genes, but also causes secretion by the dying cells of proliferative signals necessary for the surviving ones to regenerate the missing or damaged tissue (Martín et al., 2017; Pinal et al., 2019). Thus, under these circumstances there is JNK-dependant compensatory proliferation.

In contrast, after generalised damage like that caused by IR, the activities of pathways associated with regeneration like JNK, Wg or JAK/STAT do not have a role in the compensation process. These pathways are indeed activated after IR or injury (McEwen and Peifer, 2005; Smith-Bolton et al., 2009; La Fortezza et al., 2016) (Figure 2), likely a reflection of their intrinsic response to damage of any kind and probably caused by the production of Reactive Oxygen Species in the damaged tissue (Santabárbara-Ruiz et al., 2015). However, their activities are inconsequential regarding size compensation: in their absence the disc restores normal size after the death of at least 35% of the cells.

We do not know the causes behind the differential response to localised and to generalised damage. One could speculate that in the case of localised damage there might be a high local concentration of secreted signals around the damaged region, perhaps due to high levels of ROS, known to stimulate JNK activity (Santabárbara-Ruiz et al., 2015). In generalised damage dying cells are dispersed throughout the tissue and the concentration of proliferative signals may be not be sufficient to stimulate the growth of the tissue.

In the IR-treated *hh > miRHG bsk* ^*DN*^ discs we have measured the cell proliferation rate in the anterior compartment, in which there is large amount of cell death after IR, and in the posterior compartment, in which cell death is impeded and the JNK pathway supressed. We find that the anterior/posterior mitotic index ratio is not affected by IR (Figure 5), indicating that the size compensation process does not rely on increasing the cell proliferation rate of surviving cells. Strictly speaking there is no compensatory proliferation in the sense of a special wave of cell divisions triggered by the damage.

In our view the key factor in the restoration process is the mechanism that controls overall size of the disc, which stops growth once the disc has attained the stereotyped final size. After IR the discs lose a fraction of the cells, which makes them smaller (although the alteration of size is not easy to visualize) and likely revert to an earlier developmental stage (Figures 6, 7).

We propose that generalised cell death, like that caused by IR, does not trigger a specific proliferative response; it simply rejuvenates the affected discs, which revert to an earlier developmental stage. It prolongs the length of the proliferation period, what allows the surviving cells to perform additional divisions at normal rate until the disc reaches its final stereotyped size. Size compensation after generalised damage is a developmental response mechanism.

## Materials and methods

### *Drosophila* strains

The *Drosophila* stocks used in this study were: *hh-Gal4*, *sal* ^*Epv*^ *-Gal4* (gift from J. F. de Celis, CBMSO, Madrid, Spain); *tub-Gal80* ^*ts*^ (McGuire et al., 2003); *UAS-RHGmiRNA* (Siegrist et al., 2010); *STAT-10xGFP* (Bach et al., 2007); UAS*-BskRNAi* (5680R1, National Institute of Genetics stock center, Japan); *UAS-WgRNAi* (v13351, Vienna *Drosophila* Resource Center); and *UAS-STATRNAi* (33,637)*, UAS-*DomeRNA*i* (32,860)*, UAS-GFP*, *UAS-puc2A* (98,328) and Canton wild type (all from the Bloomington *Drosophila* Stock Center).

### Immunostaining

Immunostaining was performed as described previously (Shlevkov and Morata, 2012). Images were captured with a Leica (Solms, Germany) DB5500 B confocal microscope. The following primary antibodies were used: rabbit anti-Dcp1 (Cell Signalling, antibody #9578) 1:200; mouse anti-Wingless (DSHB 4D4) 1:50; guinea pig anti-Dll (Estella et al., 2008) (gift of Dr Carlos Estella); rat anti-Ci antibody (DSHB 2A1) 1:50, rabbit anti-PH3 (Millipore 06-570) 1:100; mouse anti-Cut (DSHB 2910) 1:20; guinea pig anti-Sens (gift of Dr Hugo Bellen) 1:1,000 and rabbit anti-Sc (gift of Dr Sonsoles Campuzano) 1:2000. Fluorescently labelled secondary antibodies (Molecular Probes Alexa) were used in a 1:200 dilution. TO-PRO3 (Invitrogen) was used in a 1:600 dilution to label nuclei. All the n number stated in the text represent individual larvae of the mentioned genotypes. All experiments are replicated at least three times.

### Dcp1/TUNEL and STATGFP quantifications

To estimate the percentage of cells that undergo apoptosis after irradiation, larvae of the genotype *sal* ^*Epv*^ *-Gal4/UAS-GFP* were irradiated with a 3000R dose 4 or 5 days after egg laying. Irradiation was performed with an X-Ray machine Phillips MG102. Wing imaginal discs from these larvae were stained with Dcp1 antibody or TUNEL 4 h or 24 h after irradiation. Confocal images were processed with FIJI software to measure the area fraction of Dcp1 and TUNEL labelled in the Sal region (region defined by GFP expression).

A similar procedure was used to quantify the modification of JAK/STAT activity after irradiation, using the *STAT-10xGFP* reporter (Ayala-Camargo et al., 2013). Larvae bearing the reporter were irradiated 4 or 5 days after egg laying, and the wing discs fixed 4 h or 24 h after irradiation. Confocal images from these discs were processed with FIJI software to measure in the pouch region (normally devoid of JAK/STAT activity) the gain of function of JAK/STAT, which is not normally active in that area. The comparison of irradiated discs and non-irradiated controls allows to estimate the gain of JAK/STAT activity after X-rays.

### Pathway inhibition in posterior compartment and quantifications

To block Wg, JAK/STAT or JNK activity in posterior compartments after irradiation, we designed a cross in which males bearing one of the transgenes *UAS-WgRNAi*, *UAS-DomeRNAi*, *UAS-STATRNAi*, *UAS-BskRNAi* or *UAS-puc* were crossed to *UAS-GFP; tub-Gal80* ^*ts*^, *hh-Gal4* females. Larvae originating from these crosses were raised at 17°C and irradiated with a 3000R dose 3 days after egg laying. Irradiation was performed with an X-Ray machine Phillips MG102. Afterwards the larvae were kept at 17°C for 4 h to allow apoptosis in normal genetic conditions and then shifted to 29°C for 3 days before dissection. Non-irradiated controls were treated with the same procedure except the irradiation. Wing imaginal discs were stained with TO-PRO3. Confocal images were processed with FIJI voxel counter plugin software to measure total disc volume (TO-PRO3 staining) and posterior compartment volume (GFP staining).

### EdU incorporation and quantification

Larvae of the genotype *UAS-RHGmiRNA/UAS-GFP; tub-Gal80* ^*ts*^ *, hh-Gal4* were raised at 25°C for 3 days, then shifted to 29°C to allow expression *RHGmiRNA*, which impedes apoptosis in the posterior compartment. 24 h after the temperature shift larvae were irradiated with a 3000R dose. Wing imaginal discs were dissected 5h and 24 h after irradiation for EdU labelling. Wing imaginal discs were cultured in 1 mL of EdU labelling solution for 30 min at room temperature and subsequently fixed in 4% paraformaldehide for 30 min at room temperature. Rabbit anti-GFP (Invitrogen) 1:200 antibody was used overnight at 4°C before EdU detection to protect GFP fluorescence. EdU detection was performed according to the manufacturer instructions (Click-iT EdU Alexa Fluor 555 Imaging Kit, ThermoFisher Scientific). TO-PRO3 was used to label the entire disc area.

To quantify EdU incorporation in the anterior and posterior compartments confocal images were processed with the FIJI software to measure the area fraction of Edu staining in each compartment (delimiting the area by GFP staining).

### Cell proliferation after imaginal discs irradiation

Larvae of the genotype *hh > miRHG bsk* ^*DN*^ were irradiate with a 3000R dose. 24 h, 48 h and 72 h after IR wing imaginal discs were dissected and stain with anti-PH3 antibody. The mitotic index (MI) was obtained using the analyse particle option of FIJI software in selected areas of the A and P compartment at the different time points to calculate the MI ratio between P/A observe in the graph.

### Expression pattern of wg, Dll and the genes specifying sensory organs precursors

To find possible differences among irradiated and non-irradiated discs, it was necessary to do a rigorous timing of their expression. To study gene expression of *cut*, *sc* and *sens*, the egg-laying period in these experiments was of 3 h. First instar larvae emerging after 24 h were collected every 1 h and each batch was divided into two groups that went to different vials. 72 h later (96 h developmental time) the larvae of one vial were irradiated and those of the second served as no-irradiated control. Some larvae of the control group were dissected at that time and others 24 h later (120 h of development). Larvae of the experimental group were dissected 24 and 48 h (120h and 144 h developmental time) after IR.

When analysing the expression of *wg* and *Dll*, we used a similar methodology, although the analysis was carried out at 8 and 24 h after irradiation.

### Statistical analysis

The results were assessed and visualized with GraphPad Prism 8 software. We employed an unpaired two-tailed Student’s t-test for comparing the means of two conditions and conducted one-way ANOVA followed by Dunnett’s test for multiple comparisons. The *p*-values displayed on the graphs are denoted by asterisks as follows: 0.05 (*), 0.01 (**), 0.001 (***), and 0.0001 (****).

## Data Availability

The original contributions presented in the study are included in the article/Supplementary material, further inquiries can be directed to the corresponding author.
